# Impact of seasonal temperature variations on adverse outcomes in atrial fibrillation: comparative insights from Vigo and Murcia cohorts

**DOI:** 10.7189/jogh.16.04110

**Published:** 2026-04-17

**Authors:** Eva Soler-Espejo, María Pilar Ramos-Bratos, Inmaculada González-Bermúdez, José Miguel Rivera-Caravaca, Pablo Domínguez-Erquicia, Andrés Íñiguez-Romo, Francisco Marín, Vanessa Roldán, Sergio Raposeiras-Roubín

**Affiliations:** 1 Department of Hematology, Hospital Clínico Universitario Virgen de la Arrixaca, University of Murcia, Instituto Murciano de Investigación Biosanitaria Pascual Parrilla, Murcia, Spain; 2 Liverpool Centre of Cardiovascular Science, University of Liverpool, Liverpool John Moores University and Liverpool Heart and Chest Hospital, Liverpool, UK; 3 Department of Cardiology, Hospital Clínico Universitario Virgen de la Arrixaca, University of Murcia, Instituto Murciano de Investigación Biosanitaria Pascual Parrilla, Centro de Investigación Biomédica en Red Enfermedades Cardiovasculares, Murcia, Spain; 4 Department of Cardiology, Hospital Álvaro Cunqueiro, University of Vigo, Instituto de Investigación Biomédica Galicia Sur, Vigo, Pontevedra, Spain; 5 Faculty of Nursing, University of Murcia, IMIB-Pascual Parrilla, Centro de Investigación Biomédica en Red Enfermedades Cardiovasculares, Murcia, Spain

## Abstract

**Background:**

Ambient temperature is a key environmental factor influencing cardiovascular health. In patients with atrial fibrillation (AF), seasonal variations may affect the risk of clinical events and mortality. We aimed to assess the impact of summer heat on adverse cardiovascular events in anticoagulated AF patients.

**Methods:**

In this prospective study, we included anticoagulated AF patients from two Spanish cohorts located in cities with differing climates: Murcia and Vigo. We followed patients for two years, recording ischaemic stroke/transient ischaemic attack (TIA), major bleeding, major adverse cardiovascular events (MACE), cardiovascular death, and all-cause death.

**Results:**

We included 13 629 AF patients, with a median age of 78 years (interquartile range = 71–83), of which 53.4% were female. In both cities, summer was associated with significantly lower incidence rate ratios for MACE, cardiovascular death, and all-cause death compared with other seasons (all *P* < 0.05). In Murcia, summer was linked to a lower risk of ischaemic stroke/TIA (adjusted hazard ratio (aHR) = 0.58; 95% confidence interval (CI) = 0.35–0.96), MACE (aHR = 0.63; 95% CI = 0.45–0.89), cardiovascular death (aHR = 0.32; 95% CI = 0.19–0.56), and all-cause death (aHR = 0.41; 95% CI = 0.30–0.56) compared with winter. In Vigo, summer was associated only with a reduced risk of all-cause death (aHR = 0.65; 95% CI = 0.56–0.76) compared to winter. When comparing summers, Murcia showed an increased risk of ischaemic stroke/TIA (aHR = 3.58; 95% CI = 1.98–6.45) and MACE (aHR = 1.86; 95% CI = 1.33–2.61) compared with Vigo.

**Conclusions:**

Summer was associated with a lower risk of adverse cardiovascular events compared with winter in patients with AF living in heat-adapted cities. However, extreme heat was associated with a significantly increased cardiovascular risk.

Ambient temperature is an important determinant of cardiovascular health. Short-term exposure to extreme temperatures has been associated worldwide with increased cardiovascular morbidity and mortality, particularly among vulnerable populations [1,2]. Physiological and behavioural adaptation – such as vasodilation, sweating, air conditioning use, changes in physical activity, rest breaks, hydration, or modified outdoor exposure – may mitigate these risks [3]. Although thermoregulatory pathways likely play a central role, some mechanisms remain incompletely understood, including heterogeneous responses among patients with atrial fibrillation (AF) and variability in individual tolerance during extreme heat [4].

In Spain, the average annual temperature has increased by 0.02°C per year, with the largest rise occurring in summer [5]. Over the same period, AF prevalence has increased. AF carries an estimated lifetime risk of approximately one in three individuals [6,7] and contributes substantially to the global burden of cardiovascular disease [8,9].

Paradoxically, the warming trend in Spain and worldwide has coincided with a gradual decline in cardiovascular mortality risk, likely reflecting physiological and behavioural acclimatisation [3]. However, direct evidence on the association between seasonal temperatures and clinical outcomes in AF patients remains limited. AF patients may be particularly susceptible to thermal stress due to the interaction between cardiovascular strain and thermoregulation, especially in the presence of frailty and multimorbidity [10,11]. Age-related changes, underlying cardiac dysfunction, and dehydration vulnerability may impair compensatory responses [12], reducing tolerance to both heat and cold [13]. Heat exposure can promote dehydration and sympathetic activation, facilitating arrhythmias and increasing the risk of stroke and other cardiovascular events. Conversely, cold exposure induces vasoconstriction, elevates blood pressure and sympathetic tone, and may increase thrombogenic risk [2]. Thus, acclimatisation to local temperature conditions could modulate cardiovascular stress and adverse events in anticoagulated AF populations.

We aimed to investigate whether summer heat is associated with risks of all-cause death, cardiovascular death, ischaemic stroke and/or transient ischaemic attack (TIA), major adverse cardiovascular events (MACE), and major bleeding among anticoagulated AF patients in two Spanish cities with distinct climates: Murcia and Vigo. Murcia has hot, dry summers and mild winters, whereas Vigo has cooler temperatures and higher humidity. These contrasts provide a natural setting to evaluate how seasonal temperature may influence cardiovascular outcomes in AF patients.

## METHODS

We conducted a multicentre observational study on two cohorts of AF patients from Murcia (southeastern Spain) and Vigo (northwestern Spain).

The Murcia AF Project III included outpatients who were recently diagnosed with any type of AF, were oral anticoagulation (OAC)-naïve, and were initiated on vitamin K antagonists (VKAs) or direct-acting OACs (DOACs) at two anticoagulation clinics between 1 January 2016 and 30 November 2021. We recorded baseline sociodemographic and anthropometric characteristics, comorbidities, and concomitant therapies. All participants provided written informed consent.

We derived the Vigo cohort from the CardioCHUVI-AF registry, which included patients with confirmed AF between 1 January 2014 and 30 September 2020. Clinical, laboratory, and therapeutic data were recorded in an anonymised encoded database. Informed consent was waived due to the retrospective design.

Both cohorts included adults (≥18 years) receiving OAC therapy with VKAs or DOACs. We excluded individuals with prosthetic heart valves, rheumatic mitral valve disease, other severe valvular disease, active cancer, or prior cancer. We conducted the study in accordance with the Declaration of Helsinki and subsequent amendments.

### Brief description of meteorological conditions in Vigo and Murcia

Murcia has a semi-arid Mediterranean climate with long, hot summers and mild winters. Rainfall is scarce and irregular, mainly occurring in autumn and spring, often as short, intense events. With approximately 300 sunny days per year, it is among Spain’s warmest and driest cities. Local geography (Segura River and surrounding mountain ranges) influences its microclimate.

Vigo has an oceanic climate with mild, rainy winters and warm, relatively dry summers. High humidity and frequent precipitation (>1800 mm/y) make it one of Spain’s rainiest cities. The Atlantic Ocean and Rías Baixas estuaries moderate temperatures year-round, limiting extremes.

### Definition of seasons

We obtained seasonal temperature from the Spanish Meteorological Agency (*Agencia Estatal de Meteorología*) monthly average temperatures. Summer comprised June–August (warmest months) and winter comprised December–February (coldest months). Spring (March–May) and autumn (September–November) were transitional. No heat or cold waves were captured. To calculate the ‘seasonal average temperature’ for each city, we calculated the average temperature of the three corresponding months: June-July-August for summer, and December-January-February for winter.

### Follow-up and clinical outcomes

The follow-up period was two years. Our primary endpoints were ischaemic stroke/TIA, major bleeding according to the 2005 International Society on Thrombosis and Hemostasis criteria [14], MACE (myocardial infarction, ischaemic stroke/TIA, and/or cardiovascular death), cardiovascular death, and all-cause death. Death was classified as cardiovascular when there were unequivocal signs that the death occurred due to a cardiovascular cause [15].

We captured all information from electronic medical records, through personal interviews during patients’ routine visits to the anticoagulation clinic, and by telephone contact. The investigators identified, confirmed, and recorded all clinical outcomes without adjudication committee.

Each patient's clinical outcome was reviewed and classified by date, season, and city. For patients who did not experience any of the endpoints during follow-up, classification was based on their last follow-up date. This approach allowed us to link each patient to the corresponding ‘seasonal average temperature’ for their city.

### Statistical analysis

We presented quantitative variables as mean (standard deviation) or as median (interquartile range (IQR), and categorical variables as absolute frequencies and percentages. We assessed differences in quantitative variables between groups using the Mann-Whitney U test or Student’s *t* test, as appropriate. We used Pearson χ^2^ test to compare proportions.

We calculated incidence rates (IRs) and their Poisson 95% confidence intervals (CIs) for the primary endpoints across the different seasons and cities. We compared and expressed the IRs as IR ratios (IRRs), using the combined average of the three quarters corresponding to spring, autumn, and winter in each cohort as the reference group. To calculate the quarterly IR, we divided the total number of events across these three seasons by three and rounded to the nearest absolute number. For the comparison of summer IRs across cohorts, the Vigo cohort's summer data served as the reference.

For each endpoint, we calculated Pearson correlation coefficient (R^2^) between the city's seasonal average temperature and its IR using the cor function in *R*. We generated a correlation heatmap using the ggplot2 package, version 3.5.1.

We used adjusted Cox proportional hazards regression models to determine the independent risk of the primary endpoints by season and city where the event occurred, and by covariates reported as relevant for AF. We fit Cox regression models using the survival package, version 3.7-0. We expressed the results as adjusted hazard ratios (aHRs) with 95% CIs. We conducted survival analyses using Kaplan-Meier curves, with differences tested by the log-rank test. For visualising survival curves, we used the survminer *R* package, version 0.4.9.

We used *R*, version 4.4.0. (R Core Team, Vienna, Austria), SPSS, version 25.0 (IBM Corp., Armonk, New York, USA), and MedCalc, version 16.4.3 (MedCalc Software bvba, Ostend, Belgium) for statistical analyses. A *P*-value of <0.05 was considered statistically significant.

## RESULTS

We initially included 21 406 patients, of whom 3266 were from the MAFP-III cohort and 18 140 from the CardioCHUVI-AF cohort. After applying exclusion criteria, we included 2809 participants from the MAFP-III cohort and 10 820 participants from the CardioCHUVI-AF cohort. The final sample included 13 629 AF patients with a median age of 78 years (IQR = 71–83), of whom 53.4% were female (**Table 1**). The median CHA_2_DS_2_-VASc score was 4 (IQR = 3–4), the median CHA_2_DS_2_-VA score was 3 (IQR = 2–4), and the median HAS-BLED score was 3 (IQR = 2–3).

**Table 1:** Baseline clinical characteristics of Murcia and Vigo cohorts*

	Murcia (n = 2809)	Vigo (n = 10 820)	*P*-value
**Demographics**			
Age in years, x̄ (SD)	77 (14)	78.7 (12.1)	<0.001
Female	1525 (54.3)	5758 (53.2)	<0.001
**Type of AF**			
First diagnosed/Paroxysmal	1034 (36.8)	1558 (14.4)	<0.001
Persistent/Permanent	1775 (63.2)	9262 (85.6)	<0.001
**Comorbidities**			
Hypertension	2386 (84.9)	8132 (75.2)	<0.001
Diabetes mellitus	1084 (38.6)	2203 (20.4)	<0.001
Heart failure	588 (20.9)	1236 (11.8)	<0.001
History of stroke/TIA	580 (20.6)	787 (7.3)	<0.001
Vascular disease†	579 (20.6)	1477 (13.7)	<0.001
Renal impairment	600 (21.4)	4028 (37.2)	<0.001
Hypercholesterolemia	431 (19.4)	3608 (34.9)	<0.001
COPD/OSA	626 (22.3)	1203 (11.1)	<0.001
History of relevant bleeding	448 (16.0)	491 (4.5)	<0.001
Liver disease	119 (4.2)	260 (2.4)	<0.001
Smoking habit	623 (22.2)	433 (4.0)	<0.001
Alcoholism	229 (8.2)	1890 (17.5)	<0.001
**Concomitant treatment**			
VKAs	900 (32.0)	9393 (86.8)	<0.001
DOACs	1909 (68.0)	1427 (13.2)	<0.001
Antiarrhythmics	434 (15.5)	986 (9.1)	<0.001
ACE inhibitors	688 (24.5)	1924 (17.8)	<0.001
ARBs	1256 (44.8)	4356 (40.3)	<0.001
Beta-blockers	1851 (65.9)	4985 (46.1)	<0.001
Diuretics	1620 (57.7)	NA	NA
Oral hypoglycaemic agents	830 (29.6)	NA	NA
Insulin	249 (8.9)	NA	NA
Antiplatelet therapy	393 (14.0)	724 (6.7)	<0.001
*Aspirin alone*	249 (8.9)	668 (6.2)	<0.001
*Clopidogrel alone*	92 (3.3)	100 (0.9)	<0.001

ACE– angiotensin-converting-enzyme, AF – atrial fibrillation, ARB – angiotensin II receptor blocker, COPD – chronic obstructive pulmonary disease, DOAC – direct oral anticoagulant, NA – not available, OSA – obstructive sleep apnea, SD – standard deviation, TIA – transient ischaemic attack, VKA – vitamin K antagonist, x̄ – mean

*All values are expressed as numbers (percentages) unless specified otherwise.

†Vascular disease includes coronary artery disease and/or peripheral artery disease.

Seasonal temperature and humidity patterns differed between Murcia and Vigo (Tables S1 and S2 in the **Online Supplementary Document**). Murcia had a higher summer/winter temperature difference (14.9°C) than Vigo (8.8°C), and higher summer temperatures (25.2°C) than Vigo (19.9°C; *P* < 0.001). Differences in other seasons were <2°C. Seasonal humidity variation was similar in both cities (≈10.7%), but Vigo consistently showed higher relative humidity: >20% higher than Murcia in winter (81.1% *vs.* 59.7%) and summer (70.3% *vs.* 49.1%), and >15% higher across the remaining seasons.

### Primary endpoints across seasons and cities

There were significant differences across all primary endpoints between the Murcia and Vigo cohorts. Murcia had higher annual IRs for ischaemic stroke/TIA (n = 133; 4.74%; IR = 2.37%) compared to Vigo (n = 115; 1.06%; IR = 0.53%; *P* < 0.001), higher major bleeding (n = 154; 5.49%; IR = 2.74%) than Vigo (n = 422; 3.90%; IR = 1.95%; *P* < 0.001), higher MACE (n = 289; 10.30%; IR = 5.15%) than Vigo (n = 698; 6.45%; IR = 3.23%; *P* < 0.001), and higher cardiovascular death (n = 126; 4.49%; IR = 2.24%) than Vigo (n = 381; 3.52%; IR = 1.76%; *P* = 0.001). Conversely, Vigo had a higher annual IR of all-cause death (n = 1608; 14.86%; IR = 7.43%) than Murcia (n = 346; 12.33%; IR = 6.16%; *P* = 0.001) (Table S3 in the **Online Supplementary Document**).

In both cohorts, IRs for all endpoints were higher in the remaining quarters than in summer (**Table 2**; Table S4 in the **Online Supplementary Document**). Accordingly, summer IRRs for MACE, cardiovascular death, and all-cause death were significantly lower than those in the other seasons in both cities. During summer, Murcia also showed higher IRRs for ischaemic stroke/TIA, major bleeding, and MACE than Vigo.

**Table 2:** IRs for different outcomes by quarters of the year

	Murcia (n = 2809)					Vigo (n = 10 820)				
	**Summer**		**Rest of the year**			**Summer**		**Rest of the year**		
	**n (%)**	IR* (95% CI)	**n (%)**	Total IR* (95% CI)	Quarterly IR* (95% CI)	**n (%)**	IR* (95% CI)	**n (%)**	Total IR* (95% CI)	Quarterly IR* (95%CI)
Ischaemic stroke/TIA	24 (0.85)	0.43 (0.27–0.64)	109 (3.88)	1.94 (1.59–2.34)	0.64 (0.45–0.89)	25 (0.23)	0.12 (0.07–0.17)	90 (0.83)	0.42 (0.33–0.51)	0.14 (0.09–0.20)
Major bleeding	34 (1.21)	0.61 (0.42–0.85)	120 (4.27)	2.14 (1.77–2.55)	0.71 (0.51–0.97)	102 (0.94)	0.47 (0.38–0.57)	320 (2.96)	1.48 (1.32–1.65)	0.49 (0.41–0.60)
MACE	55 (1.96)	0.98 (0.74–1.27)	234 (8.33)	4.17 (3.65–4.74)	1.39 (1.10–1.73)	127 (1.17)	0.59 (0.49–0.70)	531 (4.91)	2.45 (2.25–2.67)	0.82 (0.70–0.95)
Cardiovascular death	17 (0.61)	0.30 (0.18–0.48)	109 (3.88)	1.94 (1.59–2.34)	0.64 (0.45–0.89)	68 (0.63)	0.31 (0.24–0.40)	313 (2.89)	1.45 (1.29–1.62)	0.48 (0.39–0.58)
All-cause death	57 (2.03)	1.02 (0.77–1.32)	289 (10.29)	5.14 (4.57–5.77)	1.71 (1.38–2.09)	245 (2.26)	1.13 (0.99–1.28)	1363 (12.6)	6.30 (5.97–6.64)	2.10 (1.91–2.30)

CI – confidence interval, IR – incidence rate, MACE – major adverse cardiovascular events, TIA – transient ischaemic attack

*Per 100 person-years.

**Figure 1:**
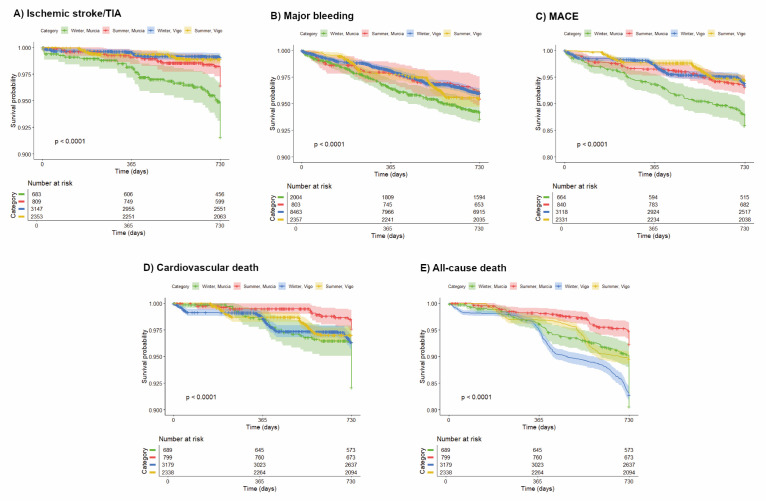
Correlations of primary endpoints between seasonal temperature and IR of each city. IR – incidence rate, MACE – major adverse cardiovascular events, TIA – transient ischaemic attack.

Across all endpoints, IRs decreased as seasonal average temperature increased in both cohorts (**Figure 1**). The strongest relationship between temperature and IR was observed for ischaemic stroke/TIA in Murcia (Table S5 in the **Online Supplementary Document**), whereas in Vigo, the strongest correlation was for MACE and the weakest for ischaemic stroke/TIA.

### Association of primary endpoints with seasonal variation by city

We adjusted the Cox models for sex, prior stroke/TIA, vascular disease, heart failure, renal disease, dyslipidemia, chronic obstructive pulmonary disease/obstructive sleep apnea, and liver disease (**Table 3**). Comparing the most extreme seasons (summer *vs.* winter), summer in Murcia was associated with lower risks of ischaemic stroke/TIA (aHR = 0.58; 95% CI = 0.35–0.96), MACE (aHR = 0.63; 95% CI = 0.45–0.89), cardiovascular death (aHR = 0.32; 95% CI = 0.19–0.56), and all-cause death (aHR = 0.41; 95% CI = 0.30–0.56), but not with a significant difference in the risk of major bleeding (aHR = 0.72; 95% CI = 0.49–1.05). In Vigo, summer was associated only with reduced all-cause death (aHR = 0.65; 95% CI = 0.56–0.76), and no significant differences were found for ischaemic stroke/TIA (aHR = 1.22; 95% CI = 0.71–2.10), major bleeding (aHR = 1.16; 95% CI = 0.92–1.44), MACE (aHR = 0.90; 95% CI = 0.72–1.13), and cardiovascular death (aHR = 0.91; 95% CI = 0.67–1.23).

**Table 3:** aHRs for different outcomes by seasons of the year

	Murcia (n = 2809)		Vigo (n = 10 820)		Murcia summer *vs.* Murcia winter (ref)		Vigo summer *vs.* Vigo winter (ref)		Murcia summer *vs.* Vigo summer (ref)	
	Summer*	Winter*	Summer*	Winter*	**aHR (95% CI)†**	***P*-value**	**aHR (95% CI)†**	***P*-value**	**aHR (95% CI)†**	***P*-value**
Ischaemic stroke/TIA	24 (0.85)	46 (1.64)	25 (0.23)	28 (0.26)	0.58 (0.35–0.96)	0.033	1.22 (0.71–2.10)	0.469	3.58 (1.98–6.45)	<0.001
Major bleeding	34 (1.21)	42 (1.50)	102 (0.94)	111 (1.03)	0.72 (0.49–1.05)	0.091	1.16 (0.92–1.44)	0.207	1.24 (0.83–1.85)	0.288
MACE	55 (1.96)	88 (3.13)	127 (1.17)	193 (1.78)	0.63 (0.45–0.89)	0.008	0.90 (0.72–1.13)	0.377	1.86 (1.33–2.61)	<0.001
Cardiovascular death	17 (0.61)	49 (1.74)	68 (0.63)	110 (1.02)	0.32 (0.19–0.56)	<0.001	0.91 (0.67–1.23)	0.528	1.14 (0.66–1.97)	0.648
All-cause death	57 (2.03)	126 (4.49)	245 (2.26)	547 (5.06)	0.41 (0.30–0.56)	<0.001	0.65 (0.56–0.76)	<0.001	0.97 (0.72–1.31)	0.848

aHR – adjusted hazard ratio, CI – confidence interval, IRR – incidence rate ratio, MACE – major adverse cardiovascular events, ref – reference, TIA – transient ischaemic attack

*Values presented as numbers (percentages).

†Adjusted by sex, stroke/transient ischaemic attack, vascular disease, heart failure, renal disease, dyslipemia, chronic obstructive pulmonary disease and liver disease.

When comparing summers between cities, Murcia summers were associated with higher risks of ischaemic stroke/TIA (aHR = 3.58; 95% CI = 1.98–6.45) and MACE (aHR = 1.86; 95% CI = 1.33–2.61) than Vigo summers. There were no significant differences in major bleeding (aHR 1.24, 95% CI 0.83-1.85), cardiovascular death (aHR = 1.14; 95% CI = 0.66–1.97), or all-cause death (aHR = 0.97; 95% CI = 0.72–1.31) (**Table 3**).

**Figure 2:**
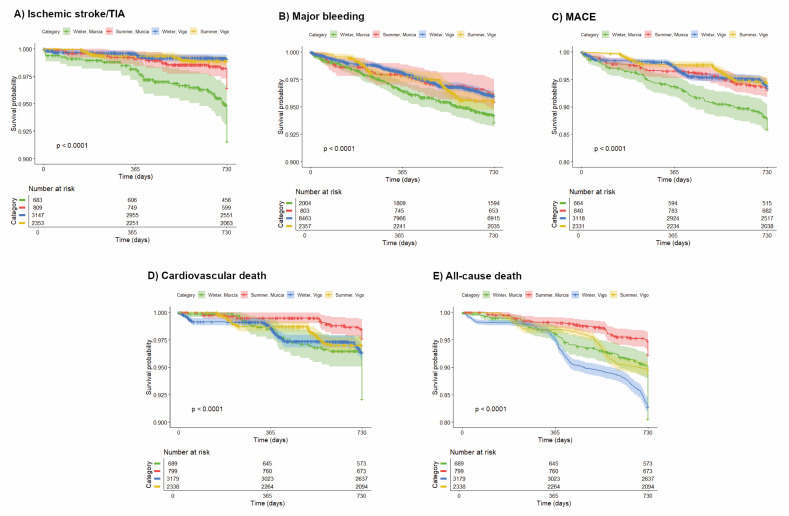
Kaplan-Meier survival curves for the primary endpoints. **Panel A.** Ischaemic stroke/TIA. **Panel B.** Major bleeding. **Panel C.** MACE. **Panel D.** Cardiovascular death. **Panel E.** All-cause death. MACE – major adverse cardiovascular events, TIA – transient ischaemic attack.

Kaplan-Meier analyses showed significant seasonal differences for all endpoints in both cities (log-rank *P* < 0.001) (**Figure 2**, Panels A–E). When comparing winters between cities, event probabilities differed for all endpoints (log-rank *P* < 0.001), except all-cause death (log-rank *P* = 0.487). When comparing summers, there were significant differences in ischaemic stroke/TIA (log-rank *P* < 0.001) and all-cause death (log-rank *P* = 0.016), but not for the remaining endpoints (Table S6 in the **Online Supplementary Document**).

## DISCUSSION

To our knowledge, we provide the broadest evaluation of the association between seasonal temperature variation and cardiovascular outcomes in anticoagulated AF patients, integrating clinical events with local climatic differences. Our findings suggest that AF risk patterns vary by season and location, and that clinical awareness may benefit from incorporating local climatic context, particularly in regions with hotter summers.

There were three key findings. First, summer was associated with a reduced incidence of MACE, cardiovascular death, and all-cause death compared with other seasons in both cohorts. Second, seasonal effects differed by city. In Murcia, summer was associated with lower risks of ischaemic stroke/TIA, MACE, cardiovascular death, and all-cause death compared with winter, whereas in Vigo, only all-cause death decreased. However, when comparing summers between cities, Murcia (with warmer summers) showed higher risks of ischaemic stroke/TIA and MACE. Third, there was an inverse association between seasonal temperature and endpoint IRs in both cohorts, with the strongest correlations for ischaemic stroke/TIA in Murcia and MACE in Vigo. To our knowledge, this is the most comprehensive assessment of seasonal temperature variations and cardiovascular events in AF patients receiving OAC therapy. Together, these findings support the possibility that local acclimatisation modulates temperature-related cardiovascular stress in AF patients.

Global warming is increasingly affecting cardiovascular health through rising temperatures and more frequent extreme weather events [12]. Spain, a climate hotspot [16], provides an ideal setting to investigate the interplay between temperature, seasonality, and cardiovascular health. We found a clear correlation between seasonal temperatures and outcomes in AF patients from two climatically distinct cities. Murcia has a warm climate with hot summers and mild winters, whereas Vigo is cooler, with milder summers and a year-round humidity >15% higher. These climatic contrasts – reflected in larger summer/winter temperature differences in Murcia (14.9°C) *vs.* Vigo (8.8°C) – underscore the different environmental exposures of the two populations and their potential influence on cardiovascular outcomes.

Temperature-related cardiovascular risk is shaped by individual susceptibility, geography, seasonality, and behavioural adaptation [13,17]. Populations living in consistently warm climates do not necessarily have higher cardiovascular risk, likely due to physiological acclimatisation and broader adaptive mechanisms [18–20], with risk often increasing during colder months [21]. Cold exposure is associated with vascular deterioration [22], increasing the risk of cardiovascular events, including acute myocardial infarction [23], heart failure [24], and worse outcomes in patients with preexisting cardiovascular disease [25]. Thermoregulatory mechanisms – such as cutaneous vasodilation, plasma volume expansion, and enhanced sweat rates – help mitigate heat stress, although their effectiveness varies with age, comorbidities, medications, and hydration status [26,27]. In Murcia, higher summer temperatures were associated with lower risks of ischaemic stroke/TIA, MACE, cardiovascular death, and all-cause death compared with winter, likely reflecting heat acclimatisation [28].

Although the relationship between seasonal temperature variation and cardiovascular risk has been widely studied in general populations [29], evidence in AF patients remains limited. Liao and colleagues conducted a multicentre study using Taiwan’s National Health Insurance Database, analysing 289 559 AF patients. They reported the highest incidence of ischaemic stroke during winter (0.33 per 100 person-months), with risk 10% higher in spring (IRR = 1.10; 95% CI = 1.07–1.13) and 19% higher in winter (IRR = 1.19; 95% CI = 1.15–1.22) compared to summer, particularly on days with temperatures <20°C [30]. In a previous study, we found that low temperatures were associated with higher risks of major bleeding (aHR = 1.03; 95% CI = 1.01–1.05) and all-cause death (aHR = 1.04; 95% CI = 1.02–1.06) [31]. Adverse events were more frequent during colder months, and winter was associated with an increased risk of adverse cardiovascular events (composite of acute coronary syndrome, acute heart failure, or cardiovascular death) (IRR = 3.78; 95% CI = 2.37–6.28) and all-cause death (IRR = 1.46; 95% CI = 1.16–1.84) compared to summer. Consistently, we found a significant reduction in IRRs for MACE, cardiovascular death, and all-cause death in summer compared with other seasons in both Murcia and Vigo. While other studies exist, most focus on winter-related new-onset AF rather than the impact of seasonal temperature variation on outcomes in anticoagulated AF patients [32–34].

A potential explanation for our findings is that Murcia’s consistently warm climate may enhance heat-related adaptive responses, leading to a more pronounced reduction in cardiovascular events than in Vigo’s milder climate. This adaptation, driven by chronic heat exposure, might promote plasma volume expansion, earlier sweating onset, and reduced sympathetic overactivation, collectively limiting atrial stretch, autonomic imbalance, and dehydration-related hemoconcentration [27,28], which could otherwise exacerbate thromboembolic risk. Conversely, in Vigo, higher summer temperatures were associated only with a reduced risk of all-cause death, suggesting that less intense summers may attenuate the benefits observed in Murcia.

Nonetheless, when comparing summers between cities (average temperature difference = 5.3°C), Murcia had higher risks of ischaemic stroke/TIA and MACE than Vigo, possibly reflecting greater thermal stress and/or heatwaves. Populations accustomed to high temperatures also remain vulnerable during such events [35]. Thermoregulatory mechanisms may be compromised during extreme heat events [36], when sudden temperature spikes overwhelm physiological coping capacity, leading to increased cardiovascular events and mortality [37]. This finding is consistent with a dual climate effect: heat may be relatively protective under stable conditions but becomes a stressor during acute extremes [38]. Evidence suggests that the greatest cardiovascular risks are driven by acute heat exposure, such as heatwaves, rather than sustained high temperatures that allow gradual adaptation [39].

The role of socioeconomic development in adaptive capacity should also be considered [40], including individual socioeconomic status, air pollution, indoor environmental conditions, physical activity, and diet. Improvements in healthcare access, education, infrastructure, and health system organisation likely enhance resilience to seasonal temperature variation [2]. Clinical management may also be relevant, as adherence to holistic strategies (*e.g.* the ABC pathway) and optimal anticoagulation quality (including stable international normalised ratio control in VKA-treated patients) may stabilise cardiovascular risk and potentially mitigate heat-related vulnerability [41]. Individual behaviours can further modify exposure and risk, as people with chronic conditions often adopt adaptive measures, such as climate control, which may modulate health impacts [42]. In addition, time spent outdoors during heatwaves or cold spells may amplify or reduce cardiovascular morbidity, complicating exposure assessment in multi-city comparisons [43]. Recognising these broader determinants is essential to guide future interventions aimed at minimising adverse outcomes in AF patients, particularly in regions with substantial climate variability.

In summary, we highlight the impact of seasonal temperature variation on cardiovascular outcomes in anticoagulated AF patients. Warmer months were associated with lower rates of MACE, cardiovascular death, and all-cause death in both cities, likely reflecting heat adaptation. However, more frequent extreme heat and heatwaves in Murcia may contribute to the higher ischaemic stroke/TIA and MACE risks observed compared with Vigo. This supports a potential dual impact of climate, with lower event rates under stable conditions but higher risk during acute extremes, although this remains speculative. Our results underscore the value of accounting for local adaptation when evaluating cardiovascular risk, and the adaptation patterns observed in Murcia may offer insights for other regions facing increasing heat exposure.

Given the increasing frequency of extreme temperatures associated with climate change, integrating seasonal variability into AF management may be clinically relevant. Our findings support public health strategies and clinical decision-making that consider local climate exposure as contextual information in routine practice, including patient education on adequate hydration, closer monitoring of vulnerable patients, and consideration of seasonal temperature patterns in follow-up and anticoagulation management. Future research should evaluate long-term heat adaptation and targeted interventions to mitigate cardiovascular risks, particularly in vulnerable populations.

### Limitations

This study has several limitations. First, as we used an observational study design, we could not establish causality between seasonal temperature variation and cardiovascular outcomes. We estimated temperature exposure using city-level averages, which may not capture individual behaviours or micro-environmental conditions. Key socioeconomic determinants (*e.g.* healthcare access and socioeconomic status) and individual behaviours (*e.g.* physical activity and diet) were unavailable and may have influenced the observed associations. Additionally, we did not measure time-varying factors, including influenza circulation and adherence to integrated care strategies (*e.g.* the ABC pathway). Although we adjusted for major confounders, residual confounding due to unmeasured variables remains possible.

Second, both cohorts were predominantly urban, which may limit the generalisability of our findings to rural populations, where climate adaptation, housing conditions, and healthcare access may differ. In addition, the lack of granular geographic information (*e.g.* municipality-level data) restricted our ability to evaluate potential urban-rural differences in temperature-related health effects.

Third, we focused on AF patients receiving OAC therapy, and the results may not fully apply to patients not anticoagulated or managed with alternative treatment strategies. Moreover, the predominantly Caucasian sample may limit extrapolation to more diverse populations.

Additionally, differences between cohorts may have influenced between-city comparisons. The Murcia cohort included 2809 patients and was prospective, enrolling anticoagulated patients with newly diagnosed AF, whereas the Vigo cohort included 10 820 patients and was retrospective, capturing a broader population with established AF. Differences in AF subtype, baseline risk, and anticoagulation management may therefore have contributed to outcome differences.

Furthemore, while we examined seasonal temperature variation, we could not assess other environmental factors – such as air pollution, green space availability, extreme temperature events, and indoor *vs.* outdoor exposure – due to data constraints. These exposures may interact with temperature and influence cardiovascular outcomes, particularly in older adults, and warrant further investigation. Finally, the relatively short follow-up limits assessment of long-term adaptation to seasonal temperature changes; longer follow-up is needed to better characterise the sustained impact of climate adaptation on cardiovascular outcomes in AF patients.

## CONCLUSIONS

We found a lower incidence of MACE, cardiovascular death, and all-cause death during summer, with more pronounced reductions in Murcia, where seasonal temperature differences are greater. Additionally, summer in Murcia was associated with significantly lower risks of ischaemic stroke/TIA and MACE compared with winter. However, summer in Murcia also had higher risks of ischaemic stroke/TIA and MACE than summer in Vigo, likely reflecting greater heat intensity. The inverse correlation between seasonal temperature and adverse cardiovascular events suggests a protective association between higher summer temperatures and lower IRs, although the magnitude of this association varied across cities.

## Additional material


Online Supplementary Document
