# Supplementary material for: Impact of seasonal temperature variations on adverse outcomes in atrial fibrillation: comparative insights from Vigo and Murcia cohorts
Source: J Glob Health. 2026 Apr 17;16:04110. doi: 10.7189/jogh.16.04110 (PMC13086485; doi:10.7189/jogh.16.04110)
Supplement: Online Supplementary Document [file jogh-16-04110-s001.pdf]

Supplement to: Soler-Espejo E, Ramos-Bratos MP, González-Bermúdez I, Rivera-Caravaca JM, Domínguez-Erquicia P, Íñiguez-Romo A, Marín F, Roldán V, Raposeiras-Roubín S. Impact of seasonal temperature variations on adverse outcomes in atrial fibrillation: comparative insights from Vigo and Murcia cohorts. J Glob Health. 2026;16:04110.

Table S1. Seasonal temperature according to each city.

**Temperature (ºC) by year**	**Murcia**				**Vigo**			
	**Winter**	**Spring**	**Summer**	**Autumn**	**Winter**	**Spring**	**Summer**	**Autumn**
2013	10.3	14.8	23.8	18.2	10.6	12.9	20.4	16.8
2014	9.2	16.3	24.8	19.0	9.7	14.4	19.6	17.5
2015	11.5	16.3	26.0	17.8	12.0	14.9	20.0	16.5
2016	9.9	15.1	25.0	18.4	11.3	13.1	20.7	16.0
2017	9.5	15.9	25.8	18.3	10.2	15.4	20.1	16.2
2018	10.5	15.2	25.1	17.7	11.5	13.2	20.4	16.8
2019	11.1	15.2	25.3	18.1	11.7	14.6	19.0	16.0
2020	10.3	15.8	25.5	17.9	10.6	14.8	20.0	16.4
2021	10.7	14.8	25.1	17.8	12.0	14.3	19.1	16.1
**Mean temperature**	**10.3**	**15.5**	**25.2**	**18.1**	**11.1**	**14.2**	**19.9**	**16.5**

Table S2. Differences of temperature between cities.

Mean temperature (ºC)	Murcia	Vigo	Difference of temperatures (Murcia - Vigo)	T-test *p-value*
Winter	10.3	11.1	-0.8	0.064
Spring	15.5	14.2	1.3	0.003
Summer	25.2	19.9	5.3	< 0.001
Autumn	18.1	16.5	1.6	< 0.001

The average summer temperature in Murcia was 25.2°C, and the average winter temperature was 10.3°C, resulting in a seasonal difference of 14.9°C. In Vigo, the summer and winter averages were 19.9°C and 11.1°C, respectively, with a difference of 8.8°C. Comparing both cities, summer temperatures differ by 5.3°C ($p<0.001$), while in other seasons, the difference was less than 2°C (Supplementary Table 3). Relative humidity patterns also differed between cities. In Murcia, average humidity ranged from 59.7% in winter to 49.1% in summer, with a seasonal variation of 10.6%. Vigo exhibited a similar seasonal change (81.1% in winter and 70.3% in summer; 10.8% difference). Despite comparable within-city seasonal variations, inter-city differences were pronounced: Vigo's humidity exceeded Murcia's by 21.4% in winter and 21.2% in summer, maintaining more than 20% higher levels than Murcia in both seasons and surpassing Murcia by over 15% throughout the year.

Table S3. Incidence rate and incidence rate ratio for the different outcomes.

	**Murcia (N = 2,807)**		**Vigo (N = 10,820)**		**Murcia *vs*. Vigo**		**Vigo *vs*. Murcia**	
	**N (%)**	**Incidence rate* (95% CI)**	**N (%)**	**Incidence rate* (95% CI)**	**IRR (95% CI)**	***p-value***	**IRR (95% CI)**	***p-value***
**Ischemic stroke/TIA**	133 (4.74)	2.37 (1.98-2.81)	115 (1.06)	0.53 (0.44-0.64)	4.46 (3.45-5.77)	<0.001	0.22 (0.17-0.29)	<0.001
**Major bleeding**	154 (5.49)	2.74 (2.33-3.21)	422 (3.90)	1.95 (1.77-2.15)	1.41 (1.16-1.70)	0.004	0.71 (0.59-0.86)	<0.001
**MACE**	289 (10.30)	5.15 (4.57-5.78)	698 (6.45)	3.23 (2.99-3.47)	1.60 (1.39-1.83)	<0.001	0.63 (0.55-0.72)	<0.001
**Cardiovascular death**	126 (4.49)	2.24 (1.87-2.67)	381 (3.52)	1.76 (1.59-1.95)	1.27 (1.03-1.56)	0.020	0.78 (0.64-0.97)	0.001
**All-cause death**	346 (12.33)	6.16 (5.53-6.85)	1608 (14.86)	7.43 (7.07-7.80)	0.83 (0.74-0.93)	0.001	1.21 (1.07-1.36)	0.009

CI = confidence interval; IRR = incidence rate ratio; MACE = major adverse cardiovascular events.

**per* 100 person-years.

Table S4. IRRs for the different outcomes by quarters of the year

	**Summer Murcia *vs.* quarter of Murcia (Ref.)**		**Summer Vigo *vs.* quarter of Vigo (Ref.)**		**Summer Murcia *vs.* summer Vigo (Ref.)**	
	**IRR (95% CI)***	***P*-value**	**IRR (95% CI)***	***P*-value**	**IRR (95% CI)***	***P*-value**
Ischaemic stroke/TIA	0.67 (0.38–1.15)	0.124	0.83 (0.47–1.41)	0.504	3.70 (2.02–6.75)	<0.001
Major bleeding	0.85 (0.52–1.38)	0.489	0.95 (0.72–1.29)	0.730	1.29 (0.84–1.91)	0.211
MACE	0.71 (0.49–1.01)	0.047	0.72 (0.57–0.91)	0.004	1.67 (1.19–2.31)	0.002
Cardiovascular death	0.47 (0.25–0.86)	0.009	0.65 (0.47–0.90)	0.006	0.96 (0.53–1.66)	0.913
All-cause death	0.59 (0.42–0.83)	0.002	0.54 (0.46–0.63)	<0.001	0.89 (0.66–1.20)	0.464

CI – confidence interval, IRR – incidence rate ratio, MACE – major adverse cardiovascular events, TIA – transient ischaemic attack

**per* 100 person-years.

Table S5. Pearson correlation coefficient (R^2) of primary endpoints between seasonal temperature and incidence rate (IR) of each city.

	**Murcia**	**Vigo**
**Ischemic stroke/TIA**	-0.899	-0.445
**Major bleeding**	-0.823	-0.634
**MACE**	-0.846	-0.966
**Cardiovascular death**	-0.858	-0.757
**All-cause death**	-0.881	-0.706

Table S6. Mantel-Cox test of primary endpoints.

**Ischemic stroke/TIA.** Global log-rank <0.001			
log-rank p-value (Mantel-Cox)	Winter, Murcia	Summer, Murcia	Winter, Vigo
Summer, Murcia	<0.001		
Winter, Vigo	<0.001	<0.001	
Summer, Vigo	<0.001	<0.001	0.585
**Major bleeding.** Global log-rank <0.001			
log-rank p-value (Mantel-Cox)	Winter, Murcia	Summer, Murcia	Winter, Vigo
Summer, Murcia	0.061		
Winter, Vigo	<0.001	0.487	
Summer, Vigo	0.006	0.970	0.299
**MACE**. Global log-rank <0.001			
Log-rank p-value (Mantel-Cox)	Winter, Murcia	Summer, Murcia	Winter, Vigo
Summer, Murcia	<0.001		
Winter, Vigo	<0.001	0.713	
Summer, Vigo	<0.001	0.170	0.154
**Cardiovascular death.** Global log-rank <0.001			
log-rank p-value (Mantel-Cox)	Winter, Murcia	Summer, Murcia	Winter, Vigo
Summer, Murcia	<0.001		
Winter, Vigo	<0.001	0.056	
Summer, Vigo	<0.001	0.294	0.183
**All-cause death**. Global log-rank <0.001			
log-rank p-value (Mantel-Cox)	Winter, Murcia	Summer, Murcia	Winter, Vigo
Summer, Murcia	<0.001		
Winter, Vigo	0.487	<0.001	
Summer, Vigo	<0.001	0.016	<0.001
